# Supplementary material for: The Inhibition of NLRP3 Inflammasome and IL-6 Production by Hibiscus noldeae Baker f. Derived Constituents Provides a Link to Its Anti-Inflammatory Therapeutic Potentials
Source: Molecules. 2020 Oct 14;25(20):4693. doi: 10.3390/molecules25204693 (PMC7587372; doi:10.3390/molecules25204693)
Supplement: Supplementary file 1 [file molecules-25-04693-s001.pdf]

Supplementary material

The inhibition of NLRP3 inflammasome and IL-6 by *Hibiscus noldeae Baker f.* derived constituents provides a link to its anti-inflammatory therapeutic potentials

Jean Claude Didelot Tomani[1,2], Vedaste Kagisha[1,3], Alembert Tchinda[4], Olivia Jansen, Allison Ledoux[3], Luc Vanhamme[2], Michel Frederich[3,§], Raymond Muganga[1,§], Jacob Souopgui[2,§,*]

[1]University of Rwanda, College of Medicine and Health Sciences, School of Medicine and Pharmacy, Kigali, Rwanda

[2]Université Libre de Bruxelles, Institute for Molecular Biology and Medicine, Department of Molecular Biology, Gosselies, Belgium.

[3]University of Liège, Centre for Interdisciplinary Research on Medicines (CIRM), Laboratory of Pharmacognosy, Liège, Belgium

[4]Laboratory of Phytochemistry, Centre for Research on Medicinal Plants and Traditional Medicine, Institute of Medical Research and Medicinal Plants Studies, Yaounde, Cameroon.

[§]*Equal contribution*

[*]Correspondence to: Laboratory of Embryology & Biotechnology, DBM-IBMM/ULB, Rue des Professeurs Jeener et Brachet, 12, 6041 Gosselies, Belgium. *E-mail address:* jsouopgu@ulb.ac.be (J. Souopgui).

***Table S.1**: The ^{1}H NMR chemical shifts for the 3 purified compounds from H. noldeae (DMSO-d_6, 500 MHz).*

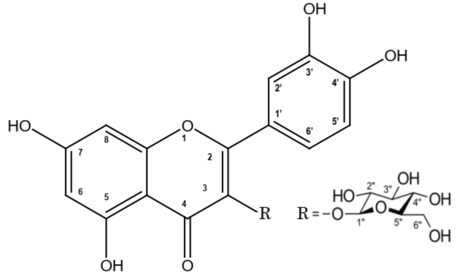 Isoquercetin		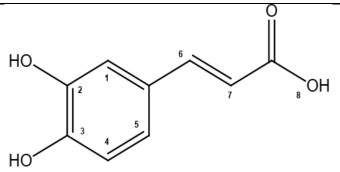 Caffeic acid		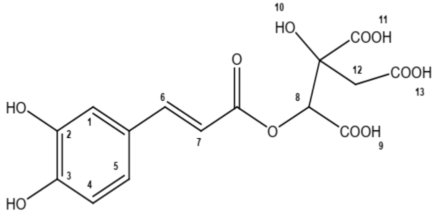 Caffeoyl-hydrocitric acid	
**Chemical shifts (ppm)**	**Protons**	**δ_H (ppm), Chemical shifts (ppm)**	**Protons**	**Chemical shifts (ppm)**	**Protons**
12.64 (1H, s)	OH-5	12.15 (1H, s)	H-8	13.3-12.35 (3H, s)	H-9, 11, 13
10.86 (1H, s)	OH-7	9.52 (1H, s)	H-3	9.64 (1H, s)	OH-2
9.72 (1H, s)	OH-3’	9.12 (1H, s)	H-2	9.18 (1H, s)	OH-3
9.15 (1H, s)	OH-4’	7.41 (1H, d)	H-6	7.57 (1H, d)	H-6
7.67 (1H, dd)	H-2’	7.02 (1H, d)	H-1	7.07 (1H, d)	H-1
7.52 (1H, d)	H-6’	6.96 (1H, dd)	H-5	7.03 (1H, dd)	H-5
6.81 (1H, d)	H-5’	6.75 (1H, d)	H-4	6.78 (1H, d)	H-4
6.40 (1H, d)	H-8	6.16 (1H, d)	H-7	6.31 (1H, d)	H-7
6.20 (1H, d)	H-6			5.24 (1H, s)	H-8
5.38 (1H, d)	H-1”			3.17 (2H, s)	H-12
5.12 (1H, s)	OH-3”				
4.84 (1H, s)	OH-4”				
4.42 (2H, s)	OH-6”				
3.65 (1H, s)	H-6”a				
3.57 (1H, m)	H-6”b				

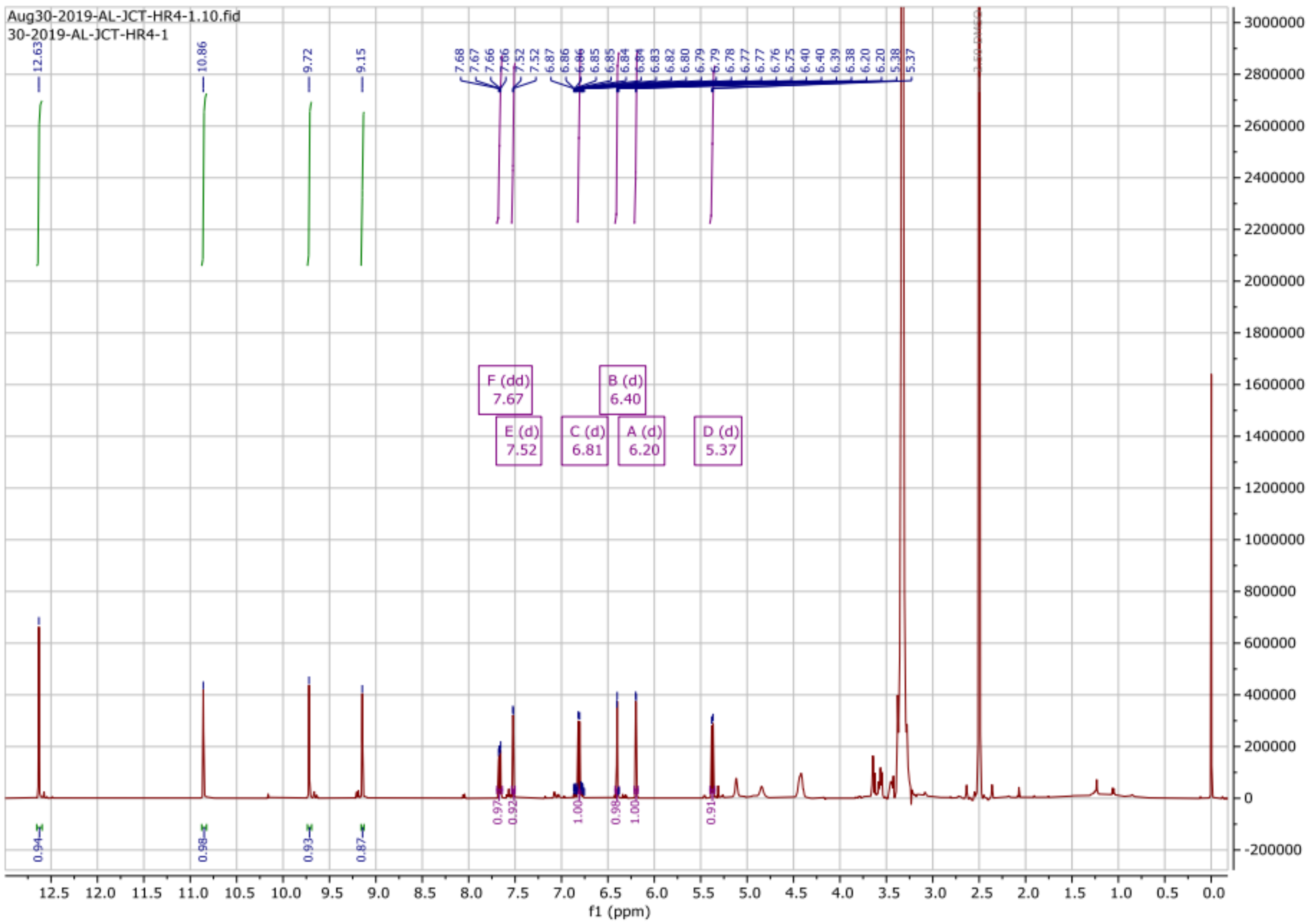

Figure S.1: ^{1}H –NMR spectrum of Quercetin-3-0-glucoside (Isoquercetin) (DMSO-d$_6$, 500 MHz)

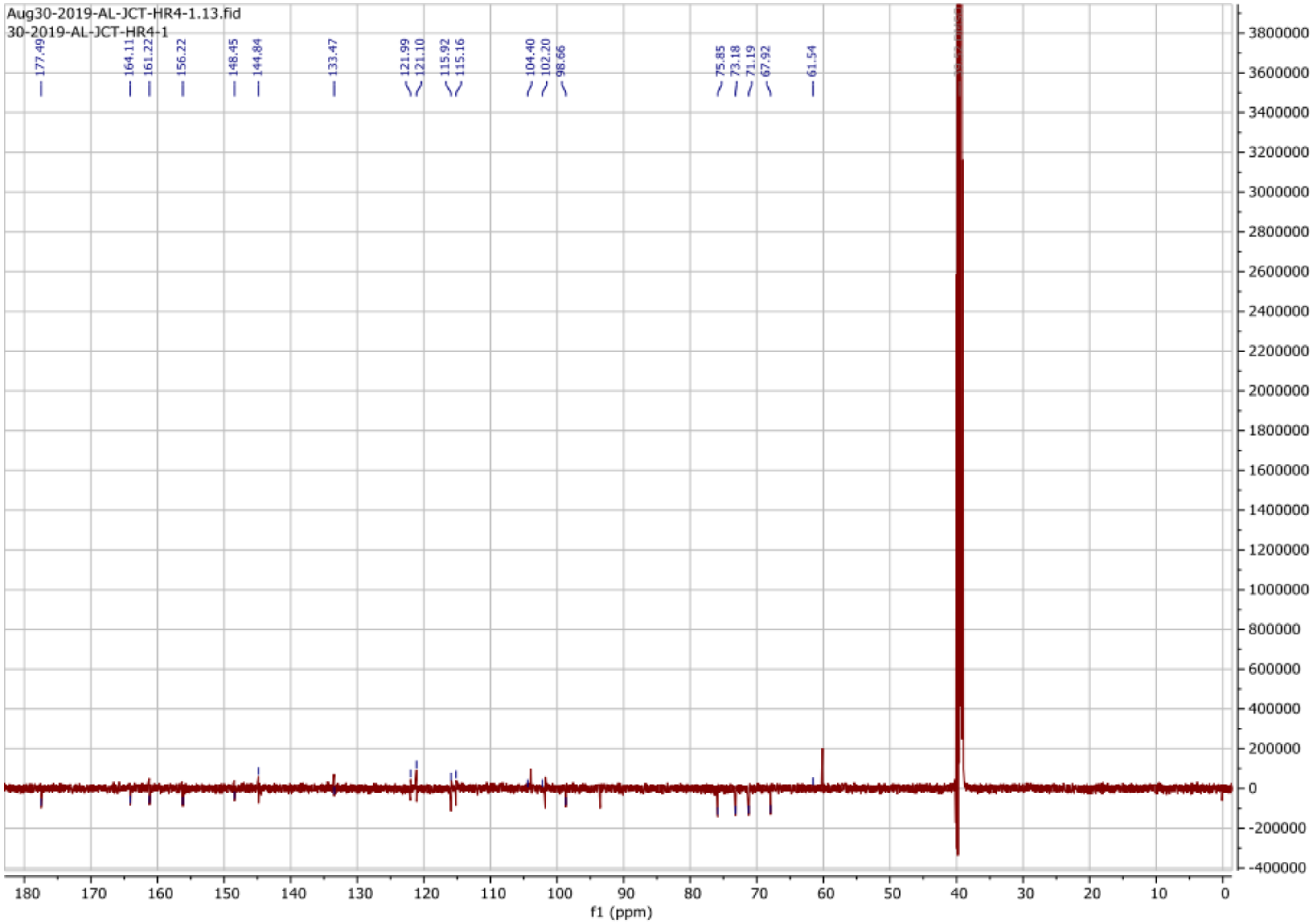

Figure S.2: ^{13}C spectrum of Quercetin-3-O-glucoside (Isoquercetin) (DMSO-d_6, 500 MHz)

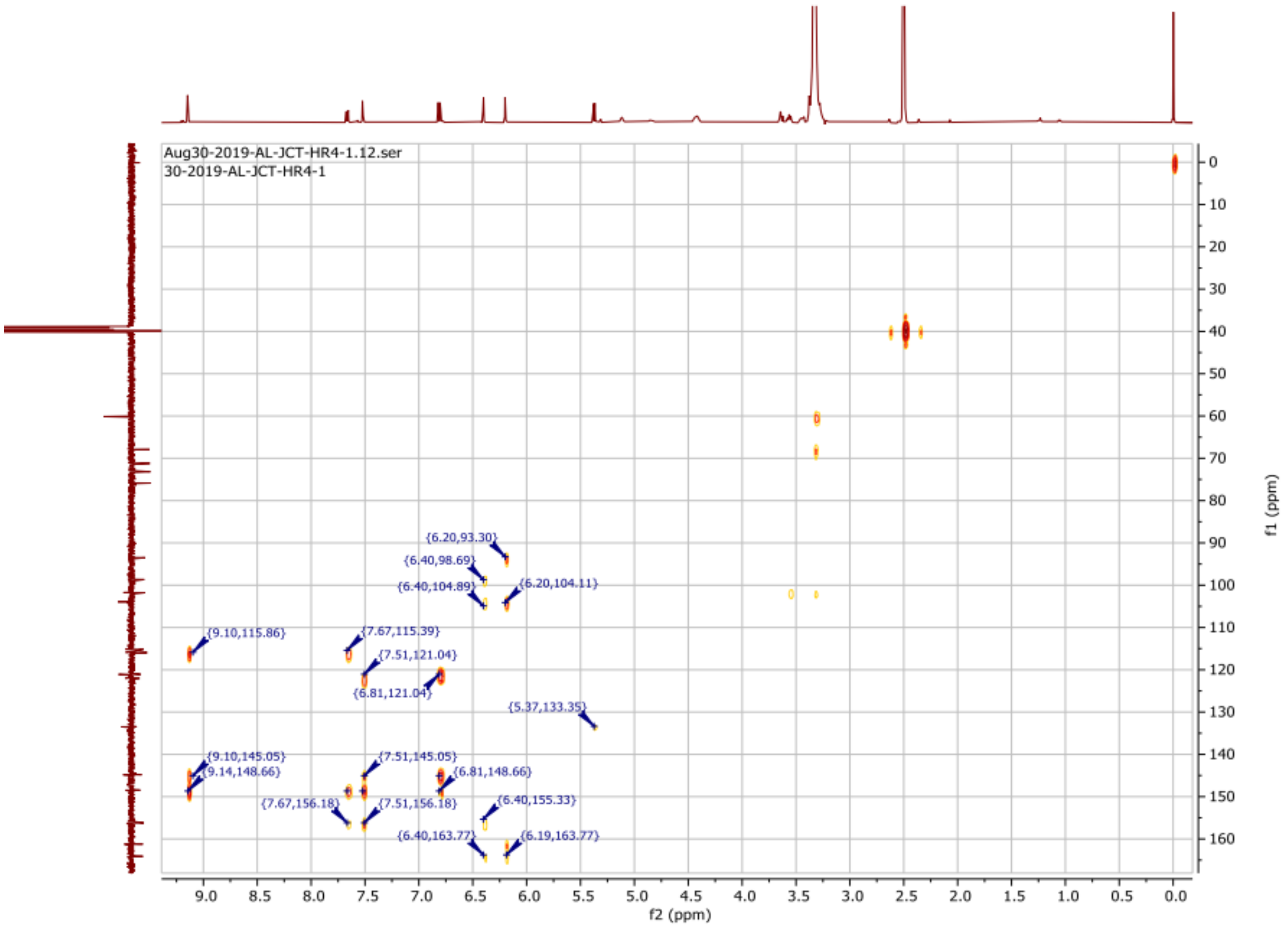

Figure S.3: 1H, 13C HMB of Quercetin-3-O-Glucoside (Isoquercetin) (DMSO-d₆, 500 MHz)

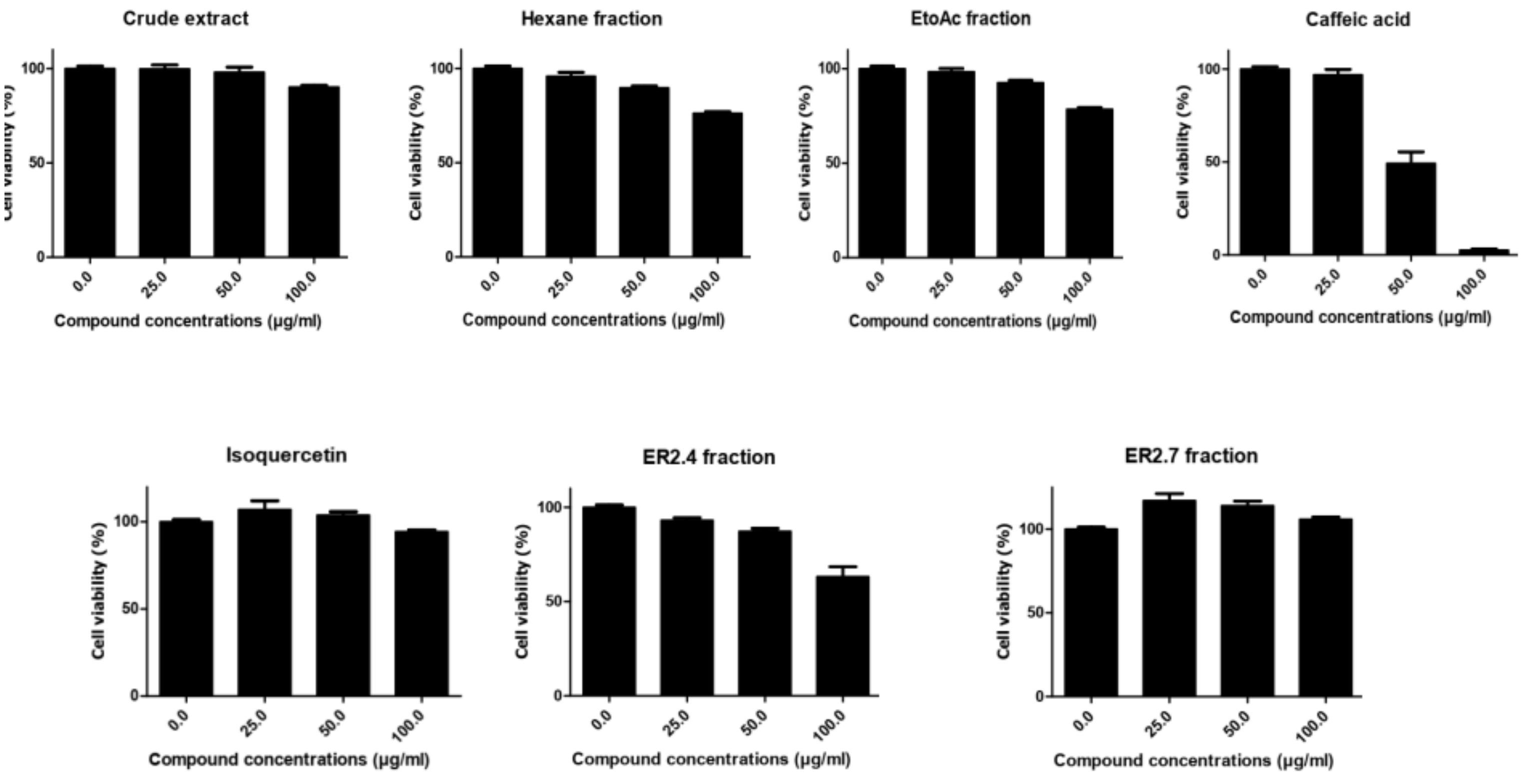

Figure S.4: Effect of different *H. noldeae*'s constituents on the THP-1 cell viability. Human leukemia THP-1 monocytes were cultured in RPMI 1640 supplemented with 10% heat-inactivated Fetal Bovine Serum (FBS), 1% glutamine and 1% of penicillin-streptomycin and maintained at 37 °C under 5% CO_2 in a humidified atmosphere. Cells were passaged at least 5 times before any experiment. Cells were then incubated with different compounds at given concentrations for 24 h at 37°C, 5%CO_2 in a humidified atmosphere. The cell viability was then evaluated by celltiter glo luminescent cell viability assay (Promega) according to the manufacturer instructions. Viability (%) = [RLU (treated cells)/ RLU (control cells)] x 100.

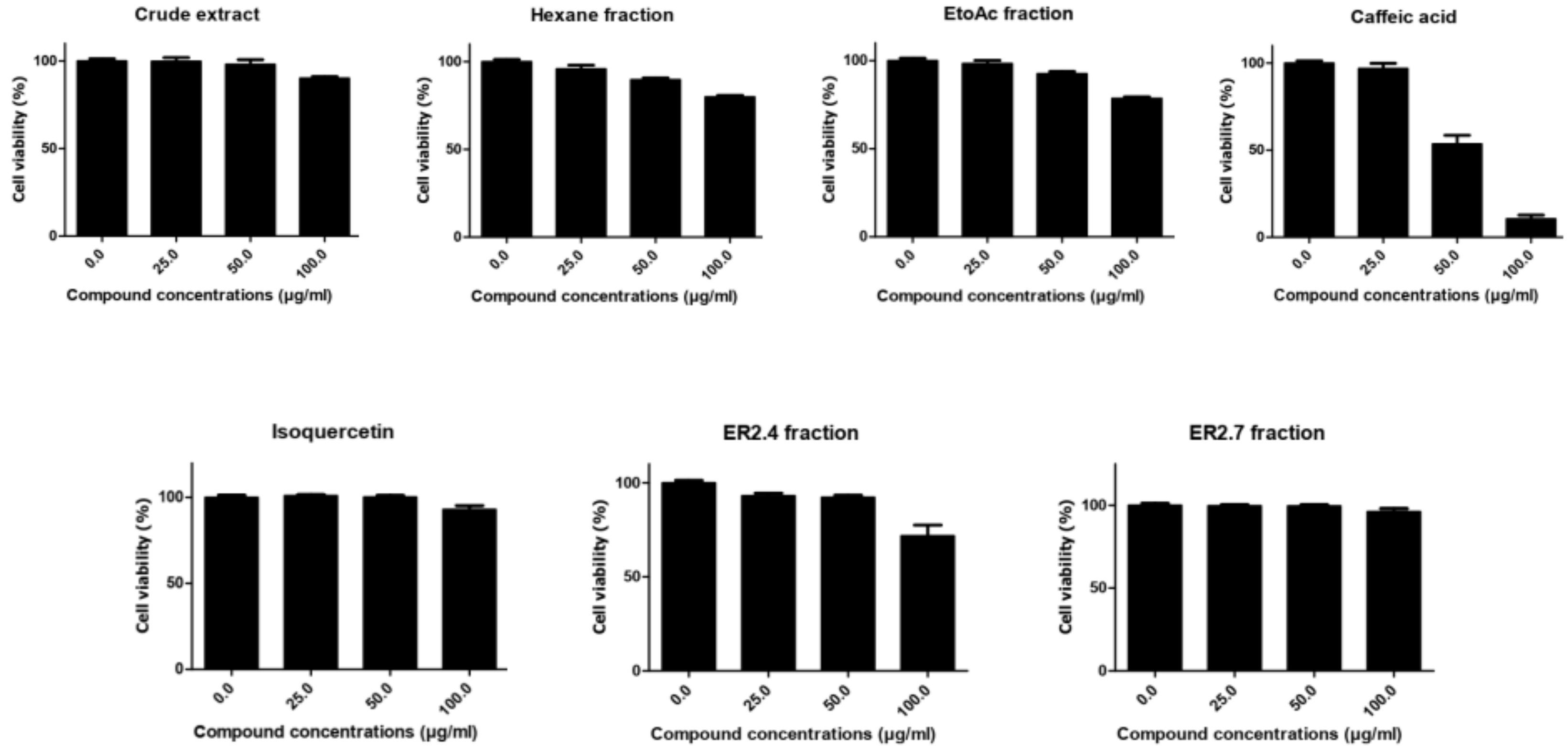

Figure S.5: Effect of different *H. noldeae*'s constituents on the RAW264.7 cell viability. RAW264.7 murine cells were cultured in DMEM supplemented with 10% heat-inactivated FBS, 1% glutamine, 1% penicillin-streptomycin and 1% Sodium pyruvate and maintained at 37 °C under 5% CO_2 in a humidified atmosphere. Cells were passaged at least 5 times, and allowed to acclimate for 24h, before any experiment. Cells were then incubated with different compounds at given concentrations for 24 h at 37°C, 5%CO_2 in a humidified atmosphere. The cell viability was then evaluated by celltiter-glo luminescent cell viability assay (Promega) according to the manufacturer instructions. Viability (%) = [RLU (treated cells)/ RLU (control cells)] x 100.

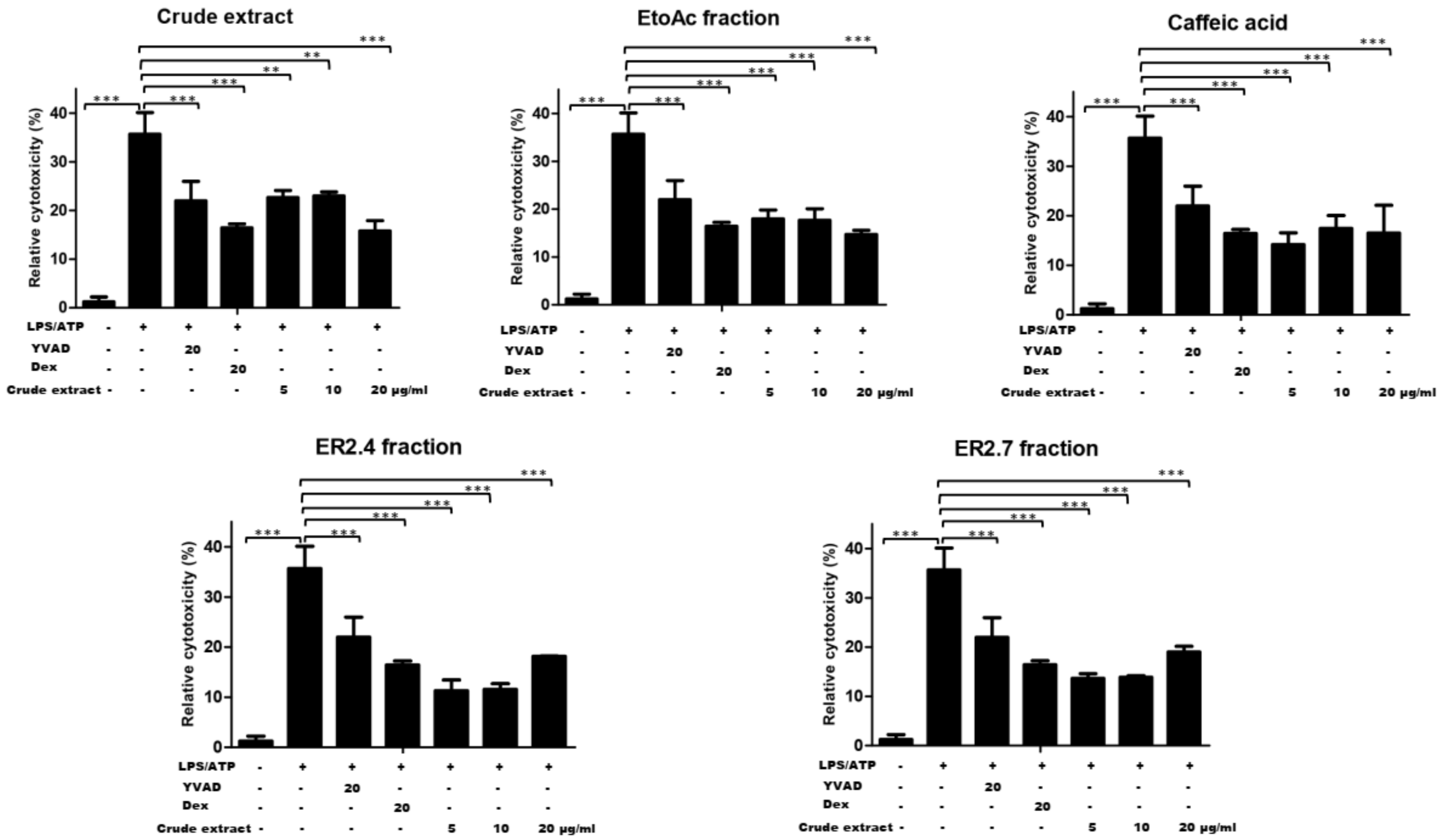

Figure S.6: Crude extract and the ethylacetate fraction as well as the caffeic acid and semi-purified fractions (ER 2.4 and ER 2.7), thereof exhibited significant inhibition of pyroptosis in THP-1 derived macrophages. PMA-differentiated cells were pretreated by corresponding compounds for 1 , and then primed by LPS (1μg/ml) for 3h. Cells were then stimulated by ATP (5mM) for additional 1 h. Samples(5μl) were collected and frozen in LDH Storage Buffer at 1:20 dilution. Samples were thawed, further diluted 5-fold and 50μl of diluted samples were added to 50μl LDH Detection Reagent. Luminescence was recorded after 60 min incubation at room temperature. The % Cytotoxicity = 100 X (Experimental LDH Release – Medium Background)/(Maximum LDH Release Control-Medium Background).
